# Supporting Primary Care Communication on Vaccination in Multilingual and Culturally Diverse Settings: Lessons from South Tyrol, Italy

**DOI:** 10.3390/epidemiologia6030050

**Published:** 2025-09-02

**Authors:** Christian J. Wiedermann, Giuliano Piccoliori, Adolf Engl

**Affiliations:** Institute of General Practice and Public Health, Claudiana—College of Health Professions, 39100 Bolzano, Italy

**Keywords:** vaccine hesitancy, health communication, communication strategies, general practitioners, cultural competence, South Tyrol, public health

## Abstract

Background: Vaccine hesitancy is a major threat to public health. As part of efforts to increase vaccine uptake, the focus is on optimizing the quality of communication among healthcare workers. Physician shortages and workloads create time constraints, making communication interventions in primary care challenging. This study aimed to propose strategies to improve communication between general practitioners and vaccine-hesitant individuals. This narrative review addresses the specific needs of general practitioners for effective communication and proposes strategies to combat vaccine hesitancy in culturally and linguistically diverse regions. Methods: Systematic searches of EMBASE and PubMed were performed using terms related to vaccine hesitancy, communication strategies, primary care, and cultural diversity. Additionally, the websites of major health organizations were searched for relevant reports and guidelines. Selection criteria were based on the relevance and quality of the selected studies. Results: The findings highlight the importance of empathy, transparency, and personalized information in communication strategies. The need for communication training and addressing policy and workload barriers for healthcare providers is significant. The proposed strategy includes regular communication skills and cultural competency workshops, language training, the development of multilingual resources, implementation of telemedicine services, and active community engagement. Conclusions: Policy recommendations advocate for increased primary care resources, support from general practitioner unions, and the integration of digital tools. These strategies are essential to improve vaccine uptake and public health outcomes by enhancing the capacity of general practitioners to effectively engage with vaccine-hesitant patients.

## 1. Introduction

Vaccine hesitancy refers to a delay in acceptance or refusal of vaccination despite the availability of vaccination services. It is influenced by factors such as confidence (trust in vaccine safety and providers), complacency (perceived need for the vaccine), and convenience (accessibility), and has been identified by the WHO as one of the top ten global health threats [1]. This phenomenon is influenced by factors such as misinformation, mistrust of health systems, and cultural beliefs [2,3]. It poses a global public health challenge by reducing herd immunity and allowing for the resurgence of preventable diseases [4,5,6,7,8]. Effective communication strategies and education are critical for addressing these issues and improving vaccine uptake worldwide [9].

In South Tyrol, Italy, vaccine hesitancy is compounded by unique linguistic and cultural diversity, with a significant portion of the population speaking German or Ladin [10]. In a recent regional survey, vaccine hesitancy was reported by 24% of the adult population, with marked variation by language group [10]. This multilingual environment creates barriers to effective communication between healthcare providers and patients [11]. In addition, cultural differences influence health beliefs and trust in medical authorities, making it difficult for GPs to effectively communicate the importance of immunization [12]. These challenges are exacerbated by time constraints and a shortage of GPs, which limit the ability to provide in-depth patient counselling and tailored communication [13]. The slow development of digital tools and support further complicates their efforts to provide consistent and accessible health information [14]. These challenges require targeted strategies to improve communication skills and resources to address vaccine hesitancy effectively in this diverse region.

This article proposes strategies to improve the communication between GPs and vaccine-hesitant patients in linguistically and culturally diverse regions. By addressing the communication challenges faced by GPs, it aims to improve immunization campaigns and increase the vaccine uptake.

## 2. Methods

Data sources for this narrative review included PubMed and EMBASE, searched in June 2024. The strategy combined keyword-based queries with manual screening of relevant organizational websites, including the World Health Organization, the European Centre for Disease Prevention and Control, and the Italian Ministry of Health. An example of the initial PubMed query used was (“vaccine hesitancy” [All Fields] OR “vaccine acceptance” [All Fields]) AND (“primary care” [All Fields] OR “general practitioners” [All Fields] OR “family medicine” [All Fields]) AND (“communication” [All Fields] OR “health communication” [All Fields] OR “patient communication” [All Fields]).

This search returned 125 results from the period January 2014 to June 2024. Relevant titles and abstracts were screened, followed by full-text review of selected publications. Additional articles were subsequently identified through manual citation tracking and artificial intelligence-supported tools: Deep Research, a custom research assistant built within the ChatGPT 4 interface (OpenAI, San Francisco, CA, USA); Consensus (Consensus Inc., New York, NY, USA); and Elicit (Ought Inc., San Francisco, CA, USA). Each platform uses natural language processing to retrieve and synthesize peer-reviewed literature relevant to user-defined queries. The search terms were iteratively refined to focus on the communication between GPs and vaccine-hesitant patients.

Inclusion criteria comprised peer-reviewed articles, reports by public health authorities, and government documents focusing on vaccine hesitancy, communication strategies in primary care, and public health interventions. Publications were excluded if they were not in English, German, or Italian; were older than 10 years unless of seminal importance; or lacked applicability to culturally and linguistically diverse settings such as South Tyrol.

Study screening and selection were performed by a single author (CJW). No formal quality scoring system was applied due to the narrative nature of this review. However, attention was paid to study design, methodological transparency, and relevance. Due to the iterative and narrative nature of the review process, no formal flowchart was established, and exact screening numbers for all databases cannot be reported. In total, 46 references were selected for inclusion in this narrative review, in addition to 4 self-citations.

## 3. Results

### 3.1. Vaccine Hesitancy in South Tyrol

In South Tyrol, vaccine hesitancy is higher among German-speaking communities than among Italian-speaking communities [15]. Mistrust of health policies, misinformation, and cultural and linguistic barriers are the key factors [10]. This is exacerbated by the region’s unique socio-political landscape, where historical tensions and linguistic diversity affect public health initiatives and vaccination rates [16]. Specific challenges include lower vaccination rates in rural areas with a higher concentration of German speakers, reflecting broader issues of education and trust in healthcare institutions [10].

GPs are a particularly important source of health information for the population of this region, particularly among German-speaking communities. This is evidenced by findings that confirm that friends and health professionals are two important sources of health-related information for the German-speaking population, indicating the special role of GPs in disseminating health information and influencing health behaviors [17].

International evidence consistently highlights the mistrust of health systems and is a key predictor of vaccine hesitancy [18]. Studies show that individuals with lower levels of education are more likely to be skeptical about vaccines because of their limited health literacy and greater susceptibility to misinformation [19]. These global patterns underscore the critical need for targeted interventions to build trust and improve education to increase the vaccine uptake.

### 3.2. General Practitioners as Trusted Sources

GPs are key to influencing public health behaviors because of their role as trusted and accessible healthcare providers [20]. They influence vaccination decisions because of their trust in and direct interaction with patients [21]. Their knowledge, beliefs, attitudes, and barriers directly affect their vaccination behavior and recommendations for patients [22]. GPs with access to scientific and official sources of information and strong communication skills are more confident in recommending vaccines, thereby positively influencing patients’ decisions. In addition, addressing barriers, such as misinformation and improving GP communication through targeted training programs, can increase vaccine uptake [23].

In South Tyrol, GPs are crucial because of the region’s linguistic and cultural diversity. They provide tailored and culturally sensitive information for various language groups. However, challenges, such as time constraints and the need for better communication skills, must be addressed. Strengthening GPs through training and resources is essential to reduce vaccine hesitancy and improve public health outcomes.

### 3.3. Communication Strategies

#### 3.3.1. Overview of Effective Communication

Effective communication strategies are critical for addressing vaccine hesitancy. Details of the strategies used to enhance GP communication skills are presented in Table 1.

A systematic review by Whitehead et al. [23] identified several effective communication strategies to counter misinformation regarding vaccines and to improve their uptake. Providing factual corrections and debunking myths effectively reduce beliefs about misinformation [24]. Disseminating knowledge regarding vaccines through various media sources has increased public awareness. Humor reduces resistance, whereas scientific consensus boosts it. Preemptive warnings about misinformation reduce misperceptions.

In addition to interpersonal communication strategies, structural and policy-level interventions have also shaped vaccine acceptance. Mandatory vaccination policies, as reviewed in global assessments, can improve immunization coverage when combined with trust-building measures and ethical implementation [25]. National and regional experiences, such as Italy’s Green Pass policy, demonstrated that combining compulsion with incentives and an active offer can lead to substantial increases in vaccine uptake, especially when accompanied by clear communication and strong health system support [26]. Furthermore, social media and mass media campaigns—when aligned with public health goals and designed to foster engagement—have proven effective in increasing public understanding and confidence in vaccines [27]. These approaches highlight how organizational, policy, and communication strategies can jointly improve vaccination outcomes, particularly in complex and diverse sociocultural contexts.

#### 3.3.2. Visual Aids, Personalizing the Message, and Engaging in Motivational Interviewing

Visual aids such as charts, infographics, and videos can help convey information more effectively than verbal explanations alone [28]. In addition, sharing the stories of patients who have benefited from vaccination or have suffered from vaccine-preventable diseases can be powerful in influencing patient decisions. Personal stories and testimonials can humanize the data and make the benefits of immunization more tangible, which allows for connecting with patients on an emotional level [24].

Personalizing messages that align with patients’ values and beliefs is effective. Discussing the impact of vaccination on the family and community makes conversations more persuasive and ensures that the information is understood and personally felt, thus motivating vaccine uptake [29].

Motivational interviewing is a patient-centered technique that encourages patients to express their thoughts about vaccination. This involved open-ended questions that affirmed autonomy. Studies have shown that it significantly increases vaccine acceptance among hesitant individuals, providing clinicians with an effective tool for engaging with these patients [24,29].

#### 3.3.3. Challenges in Culturally and Linguistically Diverse Regions

GPs face unique challenges in the linguistically and culturally diverse regions of South Tyrol. The study by Ausserhofer et al. [30] on health information-seeking emphasizes the critical need for tailored communication strategies that address these specific challenges. GPs must be trained to effectively utilize face-to-face consultations to ensure that they can address the individual concerns and circumstances of each patient [31]. Personalizing the message to align with the patient’s values and beliefs ensures that the information is not only understood, but also felt at a personal level.

Barriers include mismatches between the language spoken by patients and that of their assigned GP (e.g., German-speaking patients with Italian-speaking GPs), limited availability of multilingual educational materials, and differing cultural attitudes toward vaccination, autonomy, and institutional trust. Such barriers can impair mutual understanding and reduce the effectiveness of vaccine counselling, particularly when time for patient interaction is constrained.

Cultural competence training helps GPs to understand and respect diverse backgrounds, thus providing more effective and empathetic care. This includes the development of multilingual resources tailored to the different literacy levels. Supporting digital tools and telehealth services can enhance communication with accessible and timely information, particularly in multilingual settings.

Ongoing training and professional development are essential. Workshops on advanced communication, cultural competence, and visual aids can improve the engagement of GPs with vaccine-hesitant patients. Flexible modules, including online courses and e-learning [32], fit into busy schedules and keep GPs updated on best practices.

## 4. Discussion

Effective communication addresses vaccine hesitancy by demonstrating empathy, ensuring transparency, and providing personalized information. Empathy includes understanding and acknowledging patients’ concerns, using reflective listening, and building trust through open-ended questions in a non-judgmental environment. Transparency refers to honestly communicating both the benefits and potential risks of vaccination, thereby reducing beliefs in conspiracies [33]. Medical communication should be accessible and emotionally engaging, acknowledging fear, while providing accurate information. Addressing the emotional components of vaccine hesitancy and maintaining a stable relationship with trust are critical. Di Lorenzo et al. [34] recommended using a non-elitist language to effectively reach a wider audience.

Training for GPs should address their communication needs given their limited availability and heavy workload. Flexible modules, including online courses and e-learning platforms, can fit into busy schedules. Workshops on empathy, reflective listening, cultural competence, and hands-on simulations could improve engagement with vaccine-hesitant patients. Incentives and integration of training into regular practice can further motivate family physicians to develop these essential skills.

Additionally, it is essential to provide relevant online information that is easily accessible [35]. This aligns with requests from GPs in a recent interdisciplinary and interprofessional relational coordination survey in South Tyrol, which emphasized the need for better online information access [36]. Providing online resources can help overburdened GPs quickly access accurate and up-to-date information, thereby enhancing their ability to effectively communicate with patients.

Attitudes toward vaccination are influenced by science literacy and public health trust. In this context, knowledge plays a pivotal role, not only general science literacy but also specific knowledge about vaccines, their development, and safety mechanisms. Recent research among newly licensed physicians in Italy found that while a majority could correctly identify vaccine-related facts, a non-negligible portion struggled to distinguish factual information from misinformation, especially in the context of social media narratives [37]. This suggests that even among healthcare professionals, there is a need for structured training on how to evaluate, communicate, and contextualize scientific evidence in ways that are comprehensible to the public. Enhancing the ability of GPs to communicate vaccine-related knowledge effectively and to counteract misinformation should thus be a cornerstone of future educational strategies.

Higher science literacy correlates with positive vaccine attitudes as informed individuals critically evaluate health information and share less unreliable data. Trust in public health institutions is crucial, indicating that both literacy and trust are essential in addressing vaccine hesitancy [12]. Lower levels of education and health literacy increase susceptibility to misinformation. Hurstak et al. [19] showed that improving health literacy could enhance vaccine uptake. To support GPs in South Tyrol, relevant information should be made available online with multilingual resources tailored to the region’s linguistic diversity [35]. In addition, school-based health education can significantly improve health literacy and public health outcomes.

High workloads, increased service demands, and bureaucratic requirements have led to general reluctance among GPs to adopt new communication initiatives [38]. This reluctance is often rooted in the perception of an already excessive workload and a lack of adequate reimbursement for additional services. GP unions, while advocating for better working conditions and remuneration, often prioritize the protection of union members over empowerment through the implementation of innovative healthcare practices [39]. This focus may inadvertently slow the adoption of effective communication strategies to address vaccine hesitancy.

In addition, the shortage of GPs exacerbates these problems by stretching existing physicians and limiting their ability to provide thorough and empathetic patient counselling [40]. Policies that fail to address these systemic issues contribute to the ongoing challenge of effectively communicating the importance of immunization. To overcome these barriers, it is essential to develop policies that not only support GPs in their current roles, but also incentivize the adoption of new communication practices [41]. This includes providing adequate financial compensation for additional services, reducing unnecessary bureaucratic tasks, and ensuring sufficient staffing to distribute the workload more evenly.

### Proposed Strategy

**Figure 1:**
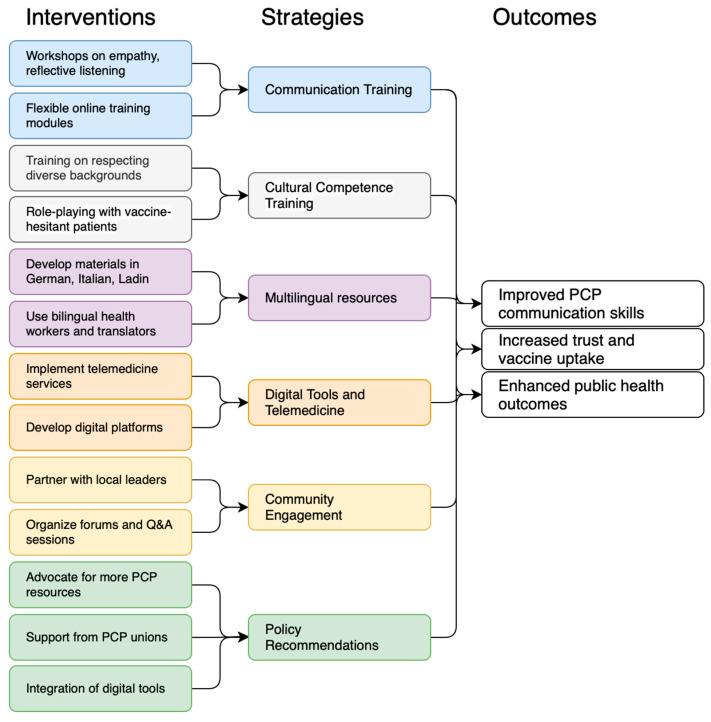
Framework for enhancing general practitioner communication to address vaccine hesitancy and improve public health outcomes in South Tyrol. Abbreviation: Q&A: questions and answers.

Addressing vaccine hesitancy and increasing vaccine uptake require comprehensive strategies [42]. This applies not only to new vaccines such as COVID-19, but also to recurring hesitancy regarding routine immunizations, including measles and influenza. These examples illustrate that communication strategies in primary care must be sustained beyond crisis contexts, with a long-term perspective on improving public trust and vaccine uptake. The proposed strategies for enhancing communication skills are summarized in Figure 1.

Understanding vaccine attitudes, intentions, and behaviors is important, with a focus on building vaccine confidence and identifying the roots of hesitancy. Effective communication techniques include using strong vaccine recommendations and presumptive formats; initiating discussions with clear, assertive statements rather than open-ended questions; and presenting vaccination as the default option [43]. Motivational interviews are an effective strategy for vaccine-hesitant parents. Specific techniques, such as open-ended questions, affirmations, reflection, and autonomy support, help build trust and encourage vaccination [33]. The role of GPs and pediatricians in these strategies is important, and cultural competence is essential to address the concerns of diverse populations [44]. Policy recommendations include the consistent application of policies, such as the dismissal of families who refuse vaccines, with a strong emphasis on ethical considerations, potential impact on attitudes toward vaccines, and trust in the medical system [43].

The key strategies for improving GP communication and vaccine uptake are as follows:

- GPs should provide clear and transparent information about vaccine safety, efficacy, and potential adverse effects, acknowledging the uncertainties in building trust. Empathic communication, including understanding and acknowledging concerns and using reflective listening, is essential. Tailoring information to a patient’s medical history, cultural background, and personal concerns makes communication more relevant and persuasive [33].
- Training programs for GPs can enhance their communication skills. Workshops should include empathic communication, reflective listening, clear information, and cultural competencies. Role-playing with actors can provide hands-on experience. To accommodate overburdened GPs, training should be flexible and accessible using online modules, short videos, and interactive e-learning schedules.
- Addressing GP workload and motivation is critical. Integrating communication training into CME credits ensures that skills are updated. Financial incentives or recognition can motivate GPs, whereas peer learning groups and mentorship programs can provide ongoing support and encouragement.
- Effective communication increases vaccine uptake and implementation of new clinical practices. Five recommendations have been made to facilitate this process. Clearly explain the rationale and benefits of the new practices, provide adequate training, use multiple communication channels, and communicate changes effectively in advance. Allowing for feedback and engagement fosters ownership and addresses concerns, helps overcome resistance, and supports implementation [45].

By adopting these strategies, family physicians can become more effective communicators, reduce vaccine hesitancy, and improve public health outcomes in their communities.

Advocating increased resources for GPs is essential for addressing the challenges faced in South Tyrol. This includes providing financial incentives for GPs involved in vaccine promotion activities and ensuring access to necessary tools and training. Increased funding and support can help manage GP workloads and integrate new communication practices effectively.

Support from GP unions is needed to improve communication skills and reduce bureaucratic burdens on GPs [46]. By advocating policies that improve working conditions and support innovative healthcare practices, GP unions can help create an environment in which GPs are motivated and able to implement effective vaccine communication strategies.

Promoting the faster adoption of telemedicine services and digital platforms can streamline processes and improve the efficiency of patient care [47]. Digital tools can help GPs better manage workloads and provide timely and accurate information to patients, enhancing their ability to address vaccine hesitancy and improving overall public health outcomes.

Effective health communication is a critical instrument to mitigate health inequalities and overcome socioeconomic disadvantage, especially during public health emergencies. Trusted communication pathways help improve understanding and acceptance of vaccine-related information, particularly among disadvantaged populations. Healthcare workers (HCWs) have a strategic role not only in administering vaccines, but also in influencing public trust and shaping vaccine-related attitudes. As frontline educators and information brokers, HCWs can strengthen intrinsic motivation toward vaccination by addressing specific concerns and promoting informed decision-making. Studies from Italy have confirmed the impact of HCW attitudes on vaccination uptake in the general population, especially through their ability to build confidence in vaccine safety and efficacy and counter hesitancy rooted in fear or mistrust [48,49,50].

In summary, this narrative review highlights tailored strategies to improve communication between general practitioners and vaccine-hesitant individuals in culturally and linguistically diverse regions. The findings underscore the need for targeted training programs that enhance interpersonal, cultural, and linguistic competencies, supported by policies that reduce workload burdens and incentivize effective communication. Moreover, the integration of telemedicine and digital platforms is essential to facilitate access to accurate vaccine information and improve the reach and efficiency of the vaccination efforts of GPs (see Supplementary Table S1 for a summary of key recommendations).

## 5. Conclusions

Effective communication strategies are a prerequisite for addressing vaccine hesitancy, especially in diverse regions, such as South Tyrol. By implementing training programs to improve communication skills and cultural competence among GPs, providing multilingual resources, and using digital tools, healthcare providers can engage with vaccine-hesitant patients better. Community engagement through collaboration with local leaders and interactive forums further supports these efforts by promoting trust and open dialogues. Policy recommendations that advocate increased GP resources, support from GP unions, and the integration of digital tools are critical for creating a supportive environment for GPs to implement these strategies. Together, these approaches can significantly improve the vaccine uptake and public health outcomes in South Tyrol.

## Figures and Tables

**Table 1:** Strategies for enhancing general practitioner communication skills.

Strategy Component	Details
Communication Techniques	
Clear and Transparent Communication	Provide straightforward information about vaccine safety, efficacy, and potential side effects.
Empathy and Reflective Listening	Understand and acknowledge patients’ concerns; establish a non-judgmental relationship.
Personalized Information	Tailor information to patients’ medical history, cultural background, and personal concerns.
Training Programs	
Workshops on Communication Skills	Focus on advanced communication techniques and cultural competence.
Cultural Competence Training	Help GPs understand and respect diverse linguistic and cultural backgrounds.
Language Training and Simulations	Provide vaccination-specific language training in German and Italian; use simulation exercises.
Flexible and Accessible Training	Offer online modules, short video tutorials, and interactive e-learning platforms.
Addressing Workload and Motivation	
Incorporate Training into Routine Work	Integrate communication training into regular meetings and CME credits.
Incentivize Participation	Offer financial incentives or recognition for completing training programs.
Support Systems	Establish peer learning groups or mentorship programs for ongoing support.
	Develop a centralized online platform where GPs can access accurate, evidence-based information about vaccines, tailored communication strategies, and updates on public health policies.
Implementation Communication	
Communication of the Intervention	Explain the reasons for adopting new practices; use various communication channels; communicate changes well in advance; encourage feedback and engagement.

## Data Availability

No new data were created.

## Supplementary Materials

The following supporting information can be downloaded at: https://www.mdpi.com/article/10.3390/epidemiologia6030050/s1. Table S1: Summary of key recommendations to support GP communication and vaccine uptake.

## Abbreviations

The following abbreviations are used in this manuscript:

CME	Continuing Medical Education
EMBASE	Excerpta Medica Database
GP	General Practitioner
PCP	Primary Care Physician
Q&A	Question and Answer
HCW	Health Care Worker

## References

[1] World Health Organization Ten Threats to Global Health in 2019. https://www.who.int/news-room/spotlight/ten-threats-to-global-health-in-2019.

[2] Galagali P.M., Kinikar A.A., Kumar V.S. (2022). Vaccine Hesitancy: Obstacles and Challenges. Curr. Pediatr. Rep..

[3] Nuwarda R.F., Ramzan I., Weekes L., Kayser V. (2022). Vaccine Hesitancy: Contemporary Issues and Historical Background. Vaccines.

[4] Pavić Ž., Kovačević E., Šuljok A. (2023). Health Literacy, Religiosity, and Political Identification as Predictors of Vaccination Conspiracy Beliefs: A Test of the Deficit and Contextual Models. Humanit. Soc. Sci. Commun..

[5] Zhao W., Russell C.M., Jankovsky A., Cannon T.D., Pittenger C., Pushkarskaya H. (2024). Information Processing Style and Institutional Trust as Factors of COVID Vaccine Hesitancy. Sci. Rep..

[6] Lazarus J.V., White T.M., Wyka K., Ratzan S.C., Rabin K., Larson H.J., Martinon-Torres F., Kuchar E., Abdool Karim S.S., Giles-Vernick T. (2024). Influence of COVID-19 on Trust in Routine Immunization, Health Information Sources and Pandemic Preparedness in 23 Countries in 2023. Nat. Med..

[7] Jacobson R.M., St Sauver J.L., Finney Rutten L.J. (2015). Vaccine Hesitancy. Mayo Clin. Proc..

[8] Haeuser E., Byrne S., Nguyen J., Raggi C., McLaughlin S.A., Bisignano C., Harris A.A., Smith A.E., Lindstedt P.A., Smith G. (2025). Global, Regional, and National Trends in Routine Childhood Vaccination Coverage from 1980 to 2023 with Forecasts to 2030: A Systematic Analysis for the Global Burden of Disease Study 2023. Lancet.

[9] Jia M. (2024). Language and Cultural Norms Influence Vaccine Hesitancy. Nature.

[10] Wiedermann C.J., Barbieri V., Plagg B., Piccoliori G., Engl A. (2024). Vaccine Hesitancy in South Tyrol: A Narrative Review of Insights and Strategies for Public Health Improvement. Ann. Ig..

[11] Slade S., Sergent S.R. (2024). Language Barrier. StatPearls.

[12] Keselman A., Arnott Smith C., Wilson A.J., Leroy G., Kaufman D.R. (2023). Cognitive and Cultural Factors that Affect General Vaccination and COVID-19 Vaccination Attitudes. Vaccines.

[13] Wang T., Tan J.-Y., Liu X.-L., Zhao I. (2023). Barriers and Enablers to Implementing Clinical Practice Guidelines in Primary Care: An Overview of Systematic Reviews. BMJ Open.

[14] Yeung A.W.K., Torkamani A., Butte A.J., Glicksberg B.S., Schuller B., Rodriguez B., Ting D.S.W., Bates D., Schaden E., Peng H. (2023). The Promise of Digital Healthcare Technologies. Front. Public Health.

[15] Barbieri V., Wiedermann C.J., Lombardo S., Ausserhofer D., Plagg B., Piccoliori G., Gärtner T., Wiedermann W., Engl A. (2022). Vaccine Hesitancy during the Coronavirus Pandemic in South Tyrol, Italy: Linguistic Correlates in a Representative Cross-Sectional Survey. Vaccines.

[16] Peterlini O., Oliveira J.C., Cardinal P. (2009). The South-Tyrol Autonomy in Italy: Historical, Political and Legal Aspects. One Country, Two Systems, Three Legal Orders—Perspectives of Evolution: Essays on Macau’s Autonomy After the Resumption of Sovereignty by China.

[17] Piccoliori G., Barbieri V., Wiedermann C.J., Engl A. (2023). Special Roles of Rural Primary Care and Family Medicine in Improving Vaccine Hesitancy. Adv. Clin. Exp. Med..

[18] Candio P., Violato M., Clarke P.M., Duch R., Roope L.S. (2023). Prevalence, Predictors and Reasons for COVID-19 Vaccine Hesitancy: Results of a Global Online Survey. Health Policy.

[19] Hurstak E.E., Paasche-Orlow M.K., Hahn E.A., Henault L.E., Taddeo M.A., Moreno P.I., Weaver C., Marquez M., Serrano E., Thomas J. (2023). The Mediating Effect of Health Literacy on COVID-19 Vaccine Confidence among a Diverse Sample of Urban Adults in Boston and Chicago. Vaccine.

[20] Oberg E.B., Frank E. (2009). Physicians’ Health Practices Strongly Influence Patient Health Practices. J. R. Coll. Physicians Edinb..

[21] MacDougall D.M., Halperin B.A., MacKinnon-Cameron D., Li L., McNeil S.A., Langley J.M., Halperin S.A. (2015). The Challenge of Vaccinating Adults: Attitudes and Beliefs of the Canadian Public and Healthcare Providers. BMJ Open.

[22] Prieto-Campo Á., García-Álvarez R.M., López-Durán A., Roque F., Herdeiro M.T., Figueiras A., Zapata-Cachafeiro M. (2022). Understanding Primary Care Physician Vaccination Behaviour: A Systematic Review. Int. J. Environ. Res. Public Health.

[23] Whitehead H.S., French C.E., Caldwell D.M., Letley L., Mounier-Jack S. (2023). A Systematic Review of Communication Interventions for Countering Vaccine Misinformation. Vaccine.

[24] Marhánková J.H., Kotherová Z., Numerato D. (2024). Navigating Vaccine Hesitancy: Strategies and Dynamics in Healthcare Professional-Parent Communication. Hum. Vaccines Immunother..

[25] Gravagna K., Becker A., Valeris-Chacin R., Mohammed I., Tambe S., Awan F.A., Toomey T.L., Basta N.E. (2020). Global Assessment of National Mandatory Vaccination Policies and Consequences of Non-Compliance. Vaccine.

[26] Stefanizzi P., Bianchi F.P., Brescia N., Ferorelli D., Tafuri S. (2022). Vaccination Strategies between Compulsion and Incentives. The Italian Green Pass Experience. Expert Rev. Vaccines.

[27] Di Mauro A., Di Mauro F., De Nitto S., Rizzo L., Greco C., Stefanizzi P., Tafuri S., Baldassarre M.E., Laforgia N. (2022). Social Media Interventions Strengthened COVID-19 Immunization Campaign. Front. Pediatr..

[28] Nuzhath T., Spiegelman A., Scobee J., Goidel K., Washburn D., Callaghan T. (2023). Primary Care Physicians’ Strategies for Addressing COVID-19 Vaccine Hesitancy. Soc. Sci. Med..

[29] Melnikow J., Padovani A., Zhang J., Miller M., Gosdin M., Loureiro S., Daniels B. (2024). Patient Concerns and Physician Strategies for Addressing COVID-19 Vaccine Hesitancy. Vaccine.

[30] Ausserhofer D., Wiedermann W., Becker U., Vögele A., Piccoliori G., Wiedermann C.J., Engl A. (2022). Health Information-Seeking Behavior Associated with Linguistic Group Membership: Latent Class Analysis of a Population-Based Cross-Sectional Survey in Italy, August to September 2014. Arch. Public Health.

[31] Alnaser F.A. (2020). Effective Communication Skills and Patient’s Health. CPQ Neurol. Psychol..

[32] Andrade M.S., Alden-Rivers B. (2019). Developing a Framework for Sustainable Growth of Flexible Learning Opportunities. High. Educ. Pedagog..

[33] Lewandowsky S., Schmid P., Habersaat K.B., Nielsen S.M., Seale H., Betsch C., Böhm R., Geiger M., Craig B., Sunstein C. (2023). Lessons from COVID-19 for Behavioural and Communication Interventions to Enhance Vaccine Uptake. Commun. Psychol..

[34] Di Lorenzo A., Stefanizzi P., Tafuri S. (2024). Are We Saying It Right? Communication Strategies for Fighting Vaccine Hesitancy. Front. Public Health.

[35] Editorial (2024). Data Access Needed to Tackle Online Misinformation. Nature.

[36] Piccoliori G., Wiedermann C.J., Barbieri V., Engl A. (2024). The Role of Homogeneous Waiting Group Criteria in Patient Referrals: Views of General Practitioners and Specialists in South Tyrol, Italy. Healthcare.

[37] Ticozzi E.M., Gaetti G., Gambolò L., Bottignole D., Di Fronzo P., Solla D., Stirparo G. (2025). Navigating Vaccine Misinformation: Assessing Newly Licensed Physicians’ Ability to Distinguish Facts from Fake News. Epidemiologia.

[38] Verhoef N.C., Blomme R.J. (2022). Burnout among General Practitioners, a Systematic Quantitative Review of the Literature on Determinants of Burnout and Their Ecological Value. Front. Psychol..

[39] van der Meer P.H. (2019). What Makes Workers Happy: Empowerment, Unions or Both?. Eur. J. Ind. Relat..

[40] Finset A., Ørnes K. (2017). Empathy in the Clinician–Patient Relationship. J. Patient Exp..

[41] van den Bussche H. (2019). Die Zukunftsprobleme der hausärztlichen Versorgung in Deutschland: Aktuelle Trends und notwendige Maßnahmen. Bundesgesundheitsbl.

[42] Jarrett C., Wilson R., O’Leary M., Eckersberger E., Larson H.J. (2015). Strategies for Addressing Vaccine Hesitancy—A Systematic Review. Vaccine.

[43] O’Leary S.T., Opel D.J., Cataldi J.R., Hackell J.M., Committee on Infectious Diseases, Committee on Practice and Ambulatory Medicine, Committee on Bioethics (2024). Strategies for Improving Vaccine Communication and Uptake. Pediatrics.

[44] Handtke O., Schilgen B., Mösko M. (2019). Culturally Competent Healthcare—A Scoping Review of Strategies Implemented in Healthcare Organizations and a Model of Culturally Competent Healthcare Provision. PLoS ONE.

[45] Albright K., Navarro E.I., Jarad I., Boyd M.R., Powell B.J., Lewis C.C. (2022). Communication Strategies to Facilitate the Implementation of New Clinical Practices: A Qualitative Study of Community Mental Health Therapists. Transl. Behav. Med..

[46] Bureaucracy Busting Concordat: Principles to Reduce Unnecessary Bureaucracy and Administrative Burdens on General Practice. https://www.gov.uk/government/publications/bureaucracy-busting-concordat-principles-to-reduce-unnecessary-bureaucracy-and-administrative-burdens-on-general-practice/bureaucracy-busting-concordat-principles-to-reduce-unnecessary-bureaucracy-and-administrative-burdens-on-general-practice.

[47] Barbosa W., Zhou K., Waddell E., Myers T., Dorsey E.R. (2021). Improving Access to Care: Telemedicine Across Medical Domains. Annu. Rev. Public Health.

[48] Stefanizzi P., Provenzano S., Santangelo O.E., Dallagiacoma G., Gianfredi V. (2023). Past and Future Influenza Vaccine Uptake Motivation: A Cross-Sectional Analysis among Italian Health Sciences Students. Vaccines.

[49] Tomietto M., Comparcini D., Simonetti V., Papappicco C.A.M., Stefanizzi P., Mercuri M., Cicolini G. (2022). Attitudes toward COVID-19 Vaccination in the Nursing Profession: Validation of the Italian Version of the VAX Scale and Descriptive Study. Ann. Ig..

[50] Mansfield L.N., Kahn B.Z., Kokitkar S., Kritikos K.I., Brantz S.N., Brewer N.T. (2024). HPV Vaccine Standing Orders and Communication in Primary Care: A Qualitative Study. Vaccine.

