# Supplementary material for: Supporting Primary Care Communication on Vaccination in Multilingual and Culturally Diverse Settings: Lessons from South Tyrol, Italy
Source: Epidemiologia (Basel). 2025 Sep 2;6(3):50. doi: 10.3390/epidemiologia6030050 (PMC12452714; doi:10.3390/epidemiologia6030050)
Supplement: Supplementary file 1 [file epidemiologia-06-00050-s001.zip › epidemiologia-3754719-supplementary material.pdf]

epidemiologia

MDPI

Supplementary Table S1. Summary of Key Recommendations to Support GP Communication and Vaccine Uptake.

Focus Area	Key Recommendations
GP Communication Skills	Offer training on empathy, cultural competence, and multilingual communication.
Workload Support	Provide incentives and reduce administrative burden.
Digital Integration	Implement telemedicine and digital tools to support communication.
Policy and System Alignment	Align public health messaging and invest in infrastructure for accessible training.
Community Engagement	Partner with trusted local figures and ensure cultural adaptation of interventions.
